# PRDM16 regulates γδT17 cell differentiation via controlling type 17 program and lipid-dependent cell fitness

**DOI:** 10.3389/fimmu.2023.1332386

**Published:** 2024-01-04

**Authors:** Jinwoo Nah, Youngjin Lee, Rho H. Seong

**Affiliations:** School of Biological Sciences, Institute of Molecular Biology and Genetics, Seoul National University, Seoul, Republic of Korea

**Keywords:** PRDM16, γδT17 cell, lipid, lipid-dependent cell fitness, psoriasis

## Abstract

γδT17 cells are a subset of γδT cells producing IL-17, which is crucial for protection against bacterial and fungal infections. It has recently been shown that γδT17 cells have enriched lipid storage and lipid metabolism. However, the regulation of γδT17 cell function and differentiation with respect to lipids remains unknown. Here, we report that PRDM16 is a critical regulator of γδT17 cell differentiation, controlling type 17 immunity gene expression program and lipid-dependent cell fitness. We demonstrated that γδT17 cells have higher lipid-dependent cell fitness, which is negatively correlated with the expression of *Prdm16*. Loss of *Prdm16* enhances the function and differentiation of γδT17 cells, and increases their fitness in lipid-rich environments. Specifically, loss of *Prdm16* exacerbates development of psoriasis in the skin, a lipid-rich organ, and *Prdm16* controls lipid-mediated differentiation of Vγ4^+^ γδT17 cells, which are the major source of IL-17 during the onset of psoriasis. Our study highlights the potential impact of PRDM16 on lipid-dependent fitness and protective immune function of γδT cells and also on the immunotherapy of psoriasis and inflammatory diseases.

## Introduction

γδT cells are a subset of T lymphocytes that can rapidly respond to antigens and play a role in both innate and adaptive immunity (1). γδT cells are also involved in tissue surveillance and protection against various types of infections (1, 2). Murine γδT cells can be further divided into two subsets: IFN-γ secreting γδT1 cells and IL-17 secreting γδT17 cells. γδT1 cells are known to show high expression of CD27 and type 1 immunity genes, such as *Tbx21* and *Eomes*, whereas γδT17 cells express low levels of CD27, and high levels of CCR6 and type 17 immunity genes, such as *Rorc* (3–5). Particularly, γδT17 cell-derived IL-17 is critical for pathogen control. It is well known that IL-23 and IL-1β, secreted by myeloid cells after bacterial infection, promote γδT17 cell expansion and activation. In addition, γδT17 cells are predominant in barrier tissues where microbe invasion occurs spontaneously, such as the lung and skin (2, 6, 7).

Murine γδT17 cells can be further segregated by the usage of their T cell receptor (TCR). γδT17 cells expressing the Vγ6 TCR chain are known to be restricted to develop in the fetal thymus (8). In addition, the generation of γδT17 cells expressing the Vγ4 TCR chain are thought to be restricted to fetal embryonic wave (8). However, γδT17 cells expressing the Vγ4 TCR chain could be developed *de novo* in the adult periphery (9, 10). It has been reported that CD27^-^ γδT17 cells can be generated from CD27^+^ CD122^-^ precursor γδT cells, and CD27^+^ CD122^+^ γδT1 cells can also be differentiated from the precursor cells (9). This peripheral differentiation is known to be strictly restricted to the Vγ4^+^ γδT cells. In addition, the induced γδT17 cells play a significant role in IL-17-mediated diseases, such as experimental autoimmune encephalomyelitis (EAE) and psoriasis (9, 10). Therefore, it is possible that natural Vγ6^+^ γδT17 cells prioritize immunosurveillance, whereas inducible Vγ4^+^ subset serves as a reservoir for further type 17 immune response, although this remains to be firmly elucidated.

Recently, it has been shown that γδT17 cell functions are closely related to lipids (11–15). Previous studies have shown an increase in γδT17 cells in High-Fat Diet (HFD) mice compared to that in Normal Chow Diet (ND) mice (11, 14). Further, a previous study reported that psoriasis can be exacerbated due to increase in γδT17 cells in HFD mice (12). Moreover, γδT17 cells show enrichment of lipid storage and metabolism (14). It has been observed that the fat layer of the dermis thickens during skin bacterial infection (16). Given that γδT17 cells play a pivotal role against bacterial infection, these results imply that the usage of lipids might be a crucial factor in the development of γδT17 cells. In this study, we show that PRDM16 is a negative regulator of type 17 immunity gene expression program and lipid-dependent cell fitness, and that this regulation by PRDM16 is fundamentally important in the generation of γδT17 cells in a lipid-rich environment such as the skin.

## Materials and methods

### Mice

*Prdm16* ^fl/fl^, *Lck*-cre, C57BL/6 mice were purchased from The Jackson Laboratory. *Prdm16* cKO mice were generated by crossing *Prdm16* ^fl/fl^ mice with *Lck*-cre mice for deletion of *Prdm16* on T cells. *Prdm16* ^fl/fl^ mice were used as control mice for comparison with *Prdm16* cKO mice (*Prdm16* ^fl/fl/^; *Lck*-cre). All mice were bred and maintained in specific pathogen-free barrier facilities at Seoul National University and were used according to protocols approved by Institutional Animal Care and Use Committees (IACUC) of Seoul National University.

### Diet intervention

Starting from 6 weeks of age, male mice were fed with a ND or HFD for 8 ~ 15 weeks, which provided 60% of energy in the form of fat (D12492; Research Diets, New Brunswick, NJ). The body weight of each mouse was monitored every week.

### IMQ-induced psoriasis model

50 mg of 5% Imiquimod (Aldara cream) was applied to the shaved back of the mice for 5 consecutive days. Modified PASI (Psoriasis Area and Severity Index) score was used to evaluate the severity of skin inflammation. Each index (erythema, scales, and thickening) was scored independently on a scale from 0 to 4: 0-none, 1-slight, 2-moderate, 3-marked, 4-maximum, and recorded every 24 h. The average score of the indexes was used to measure the severity of inflammation (scale 0-4). The area score was not considered since each mouse had the same experimental area (shaved back).

### Cell culture

All primary cells were cultured in RPMI 1640 supplemented with 10% FBS (Gibco, HyClone), 100 U/ml streptomycin and penicillin, 2-mercaptoethanol (ME).

### Isolating lymphocytes from various tissues

Liver tissues were placed in RPMI media and chopped into small pieces using scissors. Then they were thoroughly minced by using plunger of the syringe. After passing through a strainer, samples were centrifuged for 5 mins at 300 rpm in order to remove larger cells and debris. Supernatant was harvested and centrifuged for 5 mins at 1500 rpm. ACK lysing buffer was added to the cells and incubated at room temperature for 3 mins in order to remove red blood cells. After that, cells were washed and density-gradient centrifuged using 40%/70% Percoll for 20 mins at 2000 rpm, 25°C. Interfaced cells were harvested and washed twice with 1x PBS. Then prepared samples were used for further experiments.

White adipose tissues were chopped into small pieces using scissors, and digestion solution (RPMI media supplemented with 1mg/ml collagenase II, 100 μg/ml DNase I, 1% FBS, antibiotics, 2-ME) was added. Samples were incubated in a rotational shaker (200 rpm) at 37°C for 30 mins. After that, samples were passed through 70μm strainer, and RPMI media (10% FBS, antibiotics, 2-ME) was added. Cells were washed and density-gradient centrifuged using 40%/70% Percoll for 20 mins at 2000 rpm, 25°C. Interfaced cells were harvested and washed twice with 1x PBS. Then prepared samples were used for further experiments.

The ear splits were chopped into small pieces using scissors, and digestion solution (RPMI media supplemented with 1000U/ml collagenase II, 100 μg/ml DNase I, 1% FBS, antibiotics, 2-ME) was added. Samples were incubated at 37°C for 60 mins and stirred with a magnetic stirrer. After that, samples were passed through 70μm strainers, and RPMI media (10% FBS, antibiotics, 2-ME) was added. Cells were washed and density-gradient centrifuged using 40%/70% Percoll for 20mins at 2000rpm, 25°C. Interfaced cells were harvested and washed twice with 1x PBS. Then prepared samples were used for further experiments.

### Flow cytometry and cell sorting

Single-cell suspensions were prepared by passing through a strainer to get rid of cell debris. ACK lysing buffer was added to the cells and incubated at room temperature for 3mins in order to remove red blood cells. After that, 1x PBS was added up to 10 ml and washed. Cells were stained with monoclonal antibodies in various combinations in 1x PBS for 15-30mins. Flow cytometry analyses were performed using FACS Canto II (BD Bioscience), and Sony Sorter SH-800 (Sony) were used for cell sorting. Data were analyzed using FlowJo V10 software. The antibodies used are as follows: The following antibody conjugates were purchased from BD Bioscience: CD45RB (16A) - FITC; TCRβ (H57-597) - FITC; IFN-γ (XMG1.2) - APC. The following antibody conjugates were purchased from Biolegends: TCR γδ (GL3) – PerCP-Cy5.5; TCR Vγ4 (UC3-10A6) – APC. The following antibody conjugates were purchased from Invitrogen: CD27 (LG.7F9) – Biotin, APC; CD122 (TM-b1) – FITC; TCR γδ (GL3) – FITC; IL-17A (eBio17B7) -PE-Cy7; CD33 (IM7) – APC; CD4 (GK1.5) – PE-Cy7; CD8 (53-6.7) – APC eFluor780; CD3ϵ (145-2C11) – PE; TCRβ (H57-597) – APC eFluor780.

### *In vitro* γδT17 cell differentiation

Precursor γδT (CD27^+^ CD122^-^) cells from mouse spleen were isolated and cultured in RPMI media supplemented with mIL-IL-23 (5ng/ml), mIL-1β (5ng/ml), α-IFN-γ (10μg/ml), and α-CD3/CD28 Dynabeads for 3 days. For mass expansion of γδT17 cells, we generated γδT17 cells from whole spleen lymphocytes by using a previously described method by Mckenzie DR et al. (17) with little modification. Pooled spleen cells were cultured at 1 × 10^6^ cells per ml in RPMI media supplemented with mIL-23 (5ng/ml), mIL-1β (5ng/ml), α-IFN-γ (10μg/ml) in 96-well round-bottom plates coated with α-γδ TCR (clone GL3, 1μg/ml) for 3 days. Then cells were washed and cultured on new 96-well plates at 1 × 10^6^ cells per ml for a further 3 days as above without TCR stimulation. The cultured cells were harvested for further analyses.

### Intracellular staining of cytokine

For intracellular staining of cytokine, cells were treated with PMA (10 ng/ml), Ionomycin (250 ng/ml), and Brefeldin A for 4 hrs. Then intracellular cytokine staining was performed according to BD Bioscience protocol.

### Preparation of adipose tissue conditioned media

6-8-week-old male C57BL/6 mice were sacrificed and epididymal fat pads were minced into 2-3mm^3^ fragments and incubated in DMEM supplemented with 10% FBS, 100 U/ml streptomycin and penicillin for 24 hrs (3ml per fat pad). Then the media were filtered through 0.45 μm strainer and kept in -80°C until use.

### Preparation of lipid-depleted media

Lipid depletion was performed by adding fumed silica (Sigma, 10 mg/ml) to FBS (Gibco) followed by mixing overnight at 4°C. The FBS was centrifuged at 4000 rpm for 5 mins. The supernatant was sterile filtered using 0.45 μm filter. The lipid-depleted FBS was then added to RPMI 1640 media, and the media was used for further experiments.

### Differential gene expression analysis and gene set enrichment analysis

The microarray data for the gene expression of γδT1 (Eomes^+^) and γδT17 (Eomes^-^) cells (GSE85585 (5)) was used as an input for differential gene expression (DGE) analysis. The analysis was performed by using limma (18) on the galaxy platform (19). DEGs between WAT and BAT was directly obtained from GSE133500 (20) which were analyzed with DEseq2 (21). For gene set enrichment analysis (GSEA), GSEA 4.1.0 software was used.

### Quantitative RT-PCR

Total RNA was extracted from cells using TRI Reagent according to the manufacturer’s instructions (Molecular Research Center, Inc.). Equivalent quantities of total RNA were reverse transcribed with Quantitect Reverse Transcription Kit (QIAGEN). cDNAs were diluted and were analyzed by quantitative real-time PCR analysis (Applied Biosystem, StepOnePlus). The expression of each gene was normalized to *Actb* expression. The primer sets used in experiments are listed in **Supplementary Table 1**.

### Statistical analysis

For calculations of statistical significance, Prism 8 (GraphPad software) was used. Data are presented as mean ± SEM/SD and were analyzed using two-tailed Student’s t-test unless stated otherwise. P values less than 0.05 were considered to be significant.

## Results

### *Prdm16* is a potential negative regulator of lipid metabolism and differentiation of γδT17 cells

**Figure 1:**
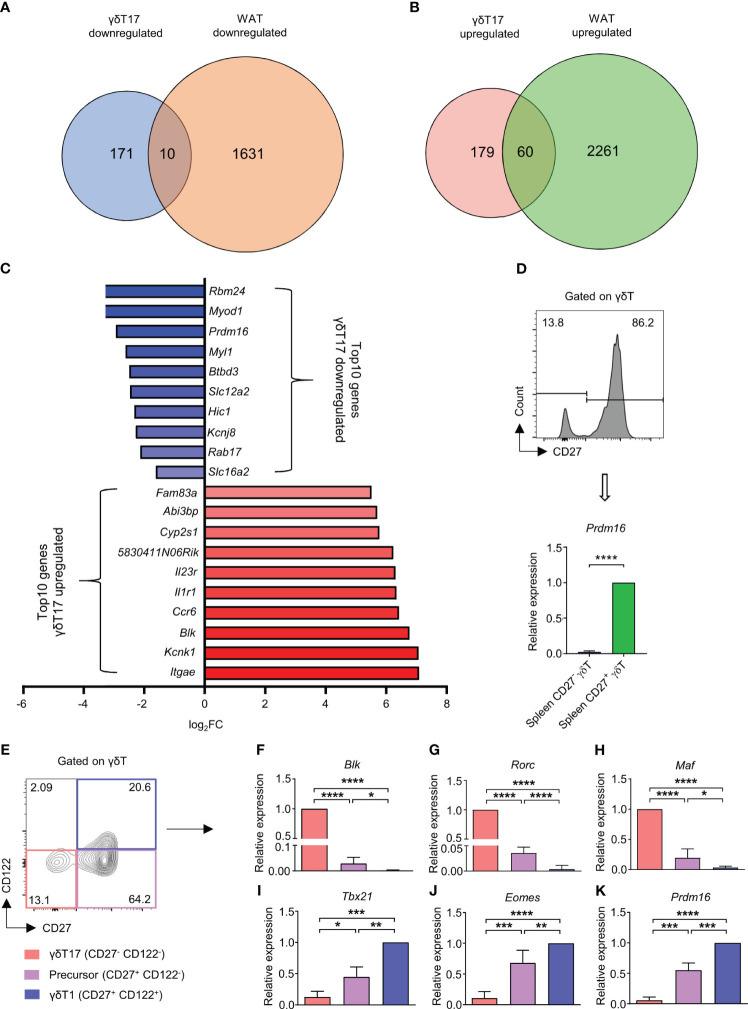
*Prdm16* is a potential negative regulator of lipid metabolism and differentiation of γδT17 cells. **(A-C)** Differentially expressed genes (DEGs) between γδT1(Eomes^+^) vs γδT17(Eomes^-^) cells and WAT vs BAT were compared. DEGs between γδT1(Eomes^+^) and γδT17(Eomes^-^) cells were obtained from GSE85585 and DEGs between WAT and BAT were obtained from GSE133500. **(A)** Down-regulated DEGs in γδT17 cells and WAT were overlapped. **(B)** Up-regulated DEGs in γδT17 cells and WAT were overlapped. **(C)** Top 10 genes were displayed among overlapped down-regulated and up-regulated genes in γδT17 cells. **(D)** Gene expression of *Prdm16* in CD27^-^ and CD27^+^ γδT cells from spleen (n= 4 per group, N=3). Data are mean ± SD. Statistical analysis was performed using Student’s *t*-test. **(E-K)** γδT1(CD27^+^ CD122^+^), Precursor (CD27^+^ CD122^-^), and γδT17(CD27^-^ CD122^-^) cells were isolated from spleen and gene expression was analyzed by qPCR. **(E)** Representative flow cytometry plot of CD27 and CD122 profile in γδT cells. **(F-K)** mRNA expression of **(F)** *Blk* (n=6 per group, N=5), **(G)** *Rorc* (n=6 per group, N=5), **(H)** *Maf* (n=6 per group, N=5), **(I)** *Tbx21* (n=3 per group, N=3), **(J)** *Eomes* (n=6 per group, N=5), **(K)** *Prdm16* (n=3 per group, N=3). Data are mean ± SD. Statistical analysis was performed using one-way ANOVA followed by Tukey’s multiple comparisons test. ns(non-significant); * P < 0.05, ** P < 0.01; *** P < 0.001; **** P < 0.0001.

High lipid content in γδT17 cells might be important for their maintenance; we assumed that γδT17 cells may have a similar lipid metabolism to white adipose cells, and lipid metabolism may play a crucial role in γδT17 cell differentiation. To identify factors regulating lipid metabolism in γδT17 cells, we compared genes differentially expressed in γδT17 cells compared to γδT1 cells with ones differentially expressed in White adipose tissue (WAT) compared to Brown adipose tissue (BAT), using publicly available microarray (5) and RNA sequencing data (20). We identified 10 downregulated genes and 60 upregulated genes through overlapping of genes in γδT17 cells and WAT (**Figures 1A, B**). Among them, the factors previously known as γδT17 cell signatures, such as *Blk*, *Ccr6*, and *Il23r* (22), were highly ranked in upregulated genes. Among downregulated genes, *Prdm16*, a crucial factor regulating differentiation of BAT and browning of WAT (23–25), was notable in γδT17 cells (**Figure 1C**). We postulated that *Prdm16* may be involved in the regulation of lipid metabolism in γδT17 cells, and that it may control the γδT17 cell differentiation as well.

We first examined the expression level of *Prdm16* in several immune cell types through ImmGen project data (26). We observed that *Prdm16* was specifically and highly expressed in γδT cells compared to other cell types (**Supplementary Figures 1A, B**). Moreover, the expression of *Prdm16* was the highest in the spleen γδT cells compared to γδT cells in other tissues (**Supplementary Figure 1C**). Between two subsets of γδT cells, CD27^+^ IFN-γ secreting γδT cells expressed *Prdm16* at considerably higher level than that of CD27^-^ IL-17 secreting γδT cells (**Figure 1D**).

Recently, γδT cells were grouped into three populations; γδT1 (CD27^+^ CD122^+^), γδT17 (CD27^-^ CD122^-^) and their precursor (CD27^+^ CD122^-^) cells (9). Indeed, we verified that isolated CD27^+^ CD122^-^ γδT cells could generate γδT1 and γδT17 cells in the presence of appropriate cytokines, such as IL-12, IL-1β, and IL-23, during the culture (**Supplementary Figure 2A**). We isolated these three populations directly from spleen and analyzed their gene expression patterns. The expression of *Blk*, *Rorc*, and *Maf* was the highest in γδT17 cells, followed by the precursor and γδT1 cells (**Figures 1E–H**). On the other hand, expression of *Tbx21* and *Eomes* was the highest in γδT1 cells, followed by the precursor and γδT17 cells (**Figures 1I, J**). Intermediate expression levels of lineage-specific factors in precursor cells indicated their multipotent characteristics. Importantly, *Prdm16* also showed graded expression among the subpopulations similar to *Tbx21* and *Eomes* (**Figure 1K**). To further investigate the relationship between γδT17 cell differentiation and *Prdm16* expression, we isolated CD27^+^ CD122^-^ precursor cells and treated with IL-1β and IL-23 to promote the development of γδT17 cells, and changes in their gene expression profiles were analyzed. Despite a short period of cytokine stimulation, the cells showed an increased level of *Il17a* and *Il23r* with an insignificant increase in the levels of *Blk* and *Rorc* (**Supplementary Figures 2B-E**), indicating a quick response of precursor γδT cells to type 17 inflammatory signals. Notably, *Prdm16* expression was decreased by cytokine stimulation (**Supplementary Figures 2F**). Taken together, these results showed that *Prdm16* is lowly expressed in γδT17 cells, suggesting its role as a potential negative regulator of lipid metabolism and γδT17 differentiation.

### High lipid content downregulates *Prdm16* expression and promotes γδT17 cell differentiation

**Figure 2:**
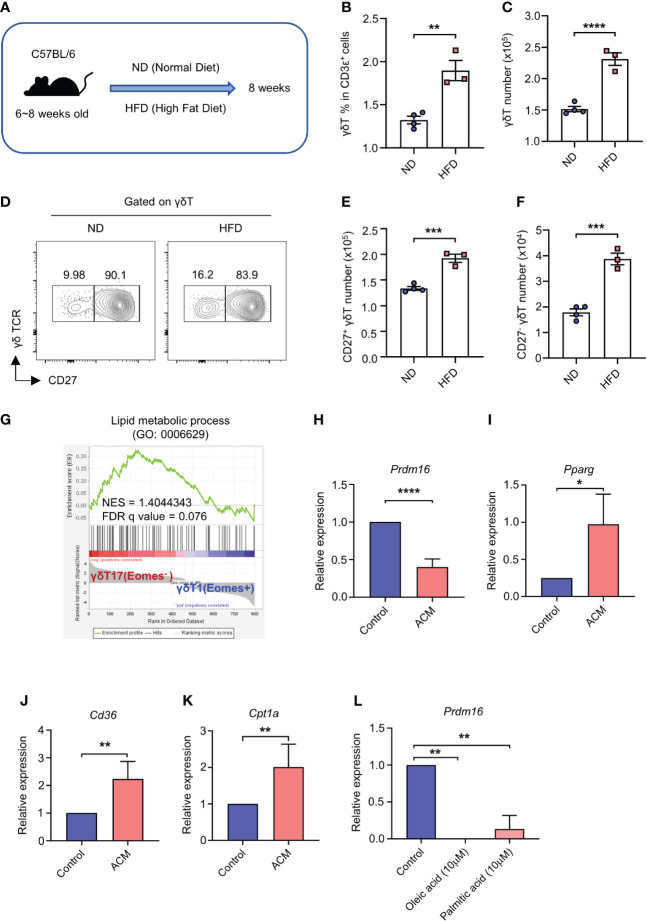
High lipid content downregulates *Prdm16* expression and promotes γδT17 cell differentiation. **(A-F)** C57BL/6 mice were fed with normal chow diet (ND) and high fat diet (HFD) for 8 weeks. Profiles of γδT cells in spleen were compared using flow cytometry. **(A)** Schematic design of the experiment. **(B)** The proportion of γδT cells within T cell population from ND/HFD mice (n=3 per group, N=1). **(C)** Total number of γδT cells in spleen from ND/HFD mice (n=3 per group, N=1). **(D)** Representative flow cytometry plot of CD27 expression in γδT cells. **(E)** Total number of CD27^+^ γδT cells in spleen from ND/HFD mice (n=3 per group, N=1). **(F)** Total number of CD27^-^ γδT cells in spleen from ND/HFD mice (n=3 per group, N=1). Data are mean ± SEM. **(G)** Gene set enrichment analysis (GSEA) between γδT1 and γδT17 cells using lipid metabolic process gene set. Input data was obtained from GSE85585. **(H-K)** Precursor γδT (CD27^+^ CD122^-^) cells were isolated and treated with adipose tissue conditioned media (ACM) for 4 hrs. Gene expression profile was analyzed by qPCR. **(H-K)** mRNA expression of **(H)** *Prdm16* (n=5 per group, N=4), **(I)** *Pparg* (n=3 per group, N=3), **(J)** *Cd36* (n=4 per group, N=3), **(K)** *Cpt1a* (n=5 per group, N=4). Data are mean ± SD. Statistical analysis was performed using Student’s *t*-test. **(L)** Precursor γδT (CD27^+^ CD122^-^) cells were isolated and treated with oleic acid (10μM) or palmitic acid (10μM) for 4 hrs. mRNA expression of *Prdm16* (n=2 per group, N=2). Data are mean ± SD. Statistical analysis was performed using one-way ANOVA followed by Tukey’s multiple comparison test. * P < 0.05; ** P < 0.01; *** P < 0.001; **** P < 0.0001.

It has been already reported that high lipids can elevate the number of γδT17 cells *in vivo* (14). According to this and our data, we speculated that lipids could impact the expression of *Prdm16* and γδT17 cell differentiation. To this end, we fed C57BL/6 mice with HFD, and analyzed the phenotypes of γδT cells (**Figure 2A**). In line with previous reports (11, 14), we observed an increased proportion and cell number of CD27^-^ γδT17 cells in the spleen of HFD mice (**Figures 2B–F**). These data indicate that high lipid content in the environment is favorable for the generation of γδT17 cells. Indeed, gene set enrichment analysis (GSEA) of microarray data (26) showed that γδT17 cells highly express genes related to lipid metabolic process (**Figure 2G**). To find out whether lipid directly regulates *Prdm16* expression in γδT cells, we isolated precursor cells and cultured them in adipose tissue conditioned media (ACM). *Prdm16* expression decreased in ACM-cultured cells compared to that in control media-cultured cells (**Figure 2H**). Moreover, genes related to adipogenesis (*Pparg*), lipid transport (*Cd36*), and fatty acid oxidation (*Cpt1a*) were increased in the ACM-cultured cells (**Figures 2I–K**). In addition, we observed that oleic acid and palmitic acid downregulated *Prdm16* expression in the precursor cells (**Figure 2L**). These data imply that lipids work as a signal for *Prdm16* downregulation, and increase the cell’s lipid adaptivity itself, which in turn could affect γδT17 cell differentiation.

### *Prdm16* deficiency enhances the differentiation and function of γδT17 cells

**Figure 3:**
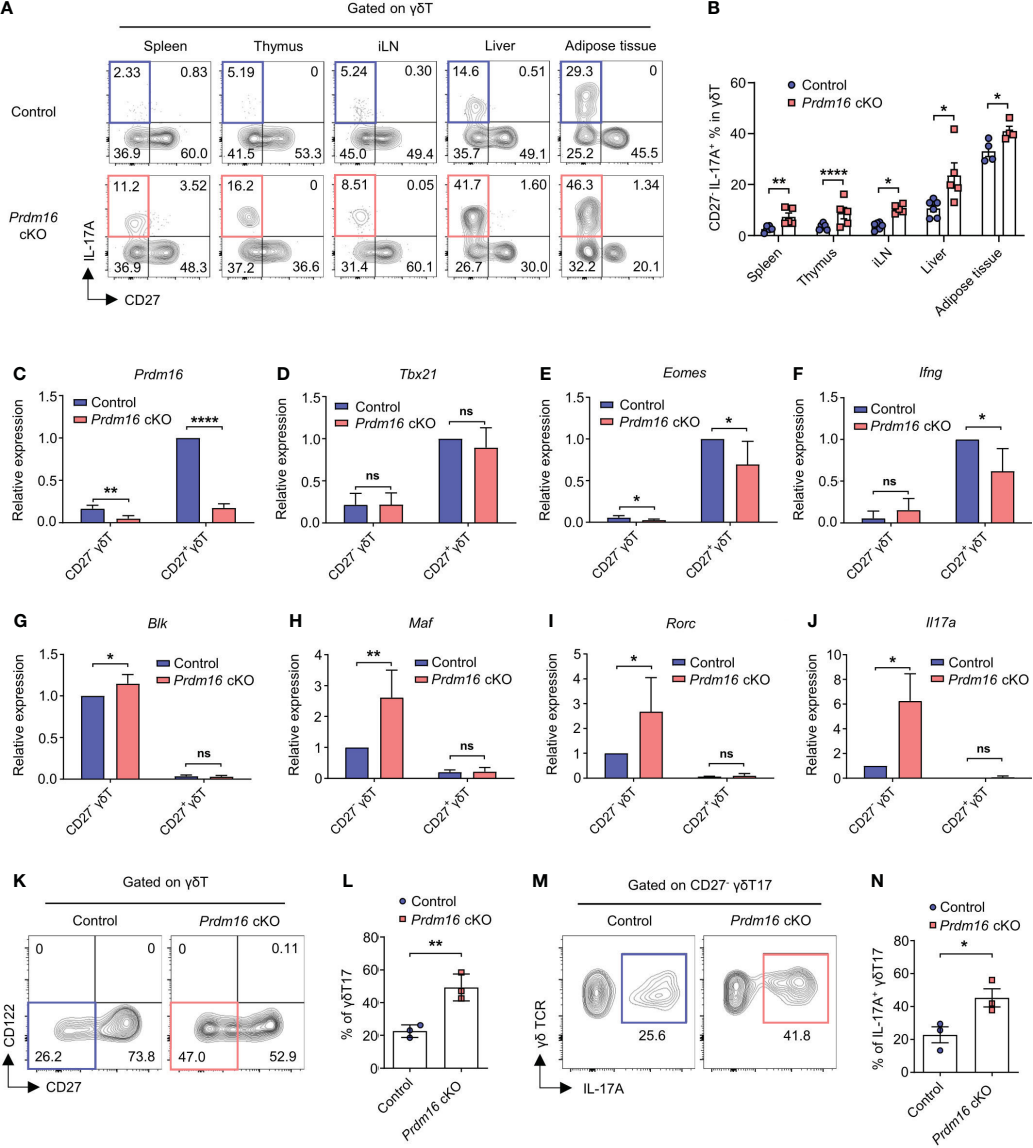
*Prdm16* deficiency enhances the differentiation and function of γδT17 cells. **(A, B)** Lymphocytes from various organs were isolated from control and *Prdm16* cKO mice and treated with PMA/Ionomycin. Profiles of γδT cells were analyzed using flow cytometry. **(A)** Representative flow cytometry plot of IL-17A and CD27 expression in γδT cells from control and *Prdm16* cKO mice. **(B)** The proportion of CD27^-^ IL-17A^+^ cells in γδT cells from control and *Prdm16* cKO mice (n=4-6 per group, N=4). **(C-J)** CD27^-^ (γδT17) and CD27^+^ (γδT1) γδT cells were isolated from the spleen of control and *Prdm16* cKO mice, and gene expression was analyzed by qPCR. mRNA expression of **(C)** *Prdm16* (n=5 per group, N=4), **(D)** *Tbx21* (n=5 per group, N=4), **(E)** *Eomes* (n=5 per group, N=4), **(F)** *Ifng* (n=4 per group, N=4), **(G)** *Blk* (n=5 per group, N=4), **(H)** *Maf* (n=5 per group, N=4), **(I)** *Rorc* (n=5 per group, N=4), **(J)** *Il17a* (n=3 per group, N=3). Data are mean ± SD. **(K-N)** Precursor γδT (CD27^+^ CD122^-^) cells were isolated from the spleen of control and *Prdm16* cKO mice, and cultured under the γδT17-driving condition. Profiles of γδT cells were analyzed using flow cytometry. **(K)** Representative flow cytometry plot of CD27 and CD122 expression in γδT cells. **(L)** The proportion of CD27^-^ γδT17 cells in γδT cells (n=3 per group, N=3). **(M)** Representative flow cytometry plot of IL-17A expression in γδT17 cells upon PMA/Ionomycin treatment. **(N)** The proportion of IL-17A^+^ CD27^-^ γδT17 cells in γδT17 cells (n=3 per group, N=3). Data are mean ± SEM. Statistical analysis was performed using Student’s *t*-test. ns(non-significant); * P < 0.05; ** P < 0.01; **** P < 0.0001.

To investigate the role of *Prdm16* in γδT17 cell differentiation, we used *Prdm16* conditional knockout (cKO) mice (*Prdm16* ^f/f^; *Lck*-cre) having T cells with loss of *Prdm16* expression. Since *Prdm16* was expressed at a very low level in most T cells other than γδT cells (**Supplementary Figures 1A, B**), we concluded the suitability of the *Lck*-cre system. Indeed, we could not find any noticeable defects in the development of T lineage cells in thymus and spleen of *Prdm16* cKO mice (**Supplementary Figures 3A, B**). Also, the proportion and number of γδT cells were comparable between control and *Prdm16* cKO mice (**Supplementary Figures 3C, D**). However, when lymphocytes from the spleen, thymus, inguinal lymph node, liver, and adipose tissue were stimulated with phorbol myristate acetate (PMA) and ionomycin, the proportion of CD27^-^ IL-17A^+^ γδT cells significantly increased in the cells of *Prdm16* cKO mice compared to that in the cells of control mice (**Figures 3A, B**). On the other hand, the proportion of CD27^+^ IFN-γ^+^ γδT cells was comparable between stimulated cells from control and *Prdm16* cKO mice (**Supplementary Figures 4A, B**). Furthermore, the proportion of IL-17A secreting cells within CD27^-^ γδT cells was remarkably increased by *Prdm16* deficiency (**Supplementary Figures 4C, D**), whereas the proportion of IFN-γ secreting cells within CD27^+^ γδT cells was comparable (**Supplementary Figures 4E, F**). To figure out whether loss of *Prdm16* enhances the function of γδT17 cells, we isolated CD27^-^ and CD27^+^ γδT cells from control and *Prdm16* cKO mice, and compared their gene expression profiles. Efficient deletion of *Prdm16* was confirmed in cKO mice (**Figure 3C**). Type 1 signature gene, including *Tbx21*, *Eomes*, and *Ifng*, were comparable or slightly decreased in *Prdm16* cKO CD27^+^ γδT1 cells (**Figures 3D–F**), whereas type 17 signature genes, including *Blk*, *Maf*, *Rorc*, and *Il17a*, were markedly increased in *Prdm16* cKO CD27^-^ γδT17 cells (**Figures 3G–J**). Moreover, when we isolated CD27^+^ CD122^-^ precursor cells and cultured them in type 17 driving condition, the proportion of differentiated CD27^-^ γδT17 cells was significantly increased by *Prdm16* deficiency (**Figures 3K, L**). The proportion of IL-17A^+^ cells within CD27^-^ γδT17 cells was also increased (**Figures 3M, N**). Collectively, these data strongly suggest that the loss of *Prdm16* strengthens differentiation and function of γδT17 cells.

### Loss of *Prdm16* increases the fitness of γδT17 cells in a high-lipid environment

**Figure 4:**
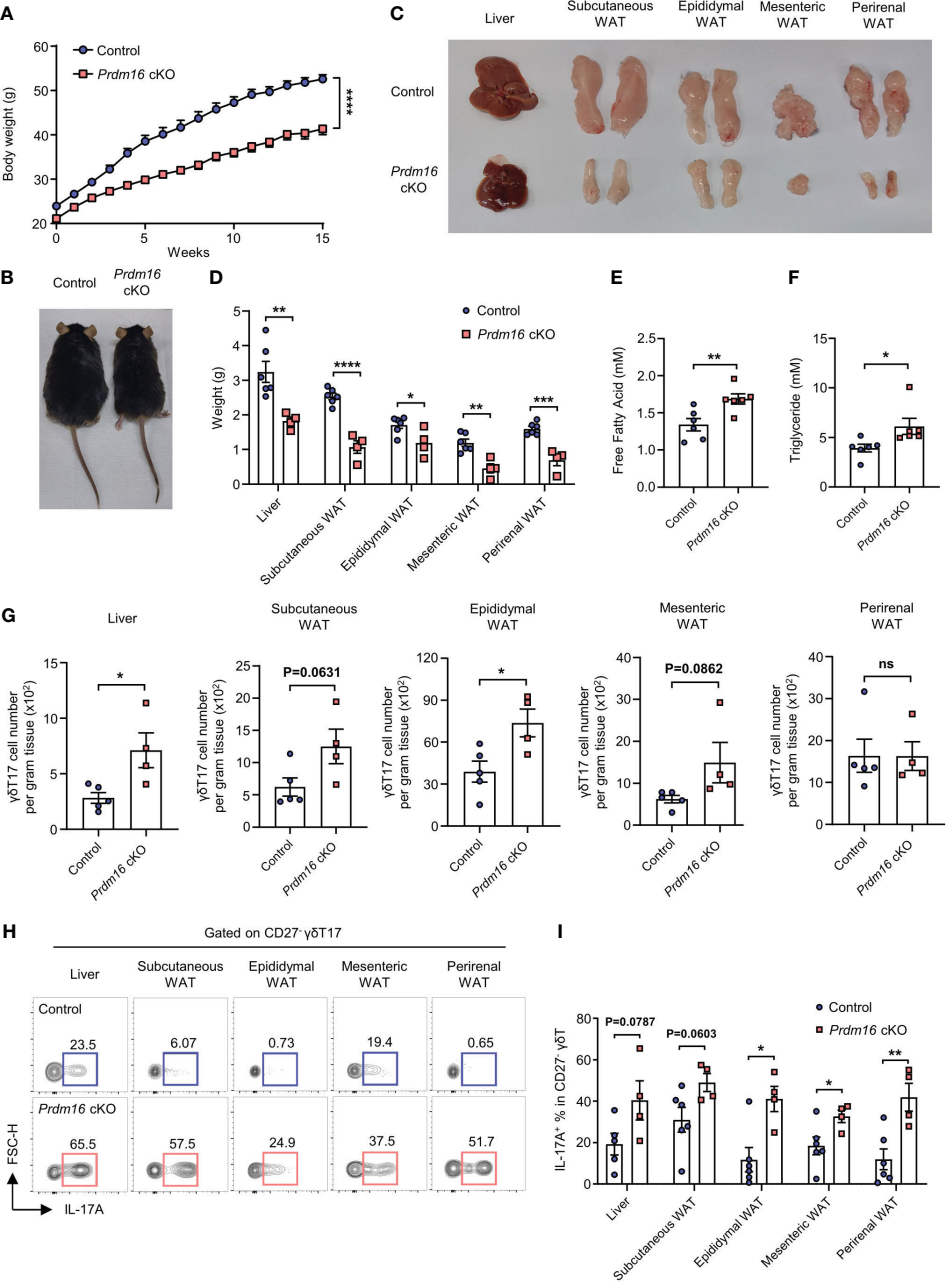
Loss of *Prdm16* increases the fitness of γδT17 cells in a high-lipid environment. **(A-I)** Control and *Prdm16* cKO mice were fed with HFD for 15 weeks. **(A)** Body weight of control and *Prdm16* cKO mice under the HFD condition (n=8-9 per group, N=3). **(B)** Body size comparison between control and *Prdm16* cKO mice. **(C)** Size of liver and various WAT from control and *Prdm16* cKO mice. **(D)** Weight of liver and various WAT from control and *Prdm16* cKO mice (n=4-6 per group, N=3). **(E)** Free fatty acid level of serum from control and *Prdm16* cKO mice (n=6 per group, N=3). **(F)** Triglyceride level of serum from control and *Prdm16* cKO mice (n=6 per group, N=3). **(G)** Total number of γδT17 cells in liver and various WAT (n=4-5 per group, N=3). **(H, I)** Lymphocytes from liver and various WAT were isolated and treated with PMA/Ionomycin. **(H)** Representative flow cytometry plot of IL-17A expression in γδT17 cells. **(I)** The proportion of IL-17A^+^ in γδT17 cells (n=4-5 per group, N=3). Data are mean ± SEM. Statistical analysis was performed using Student’s *t*-test. ns(non-significant); * P < 0.05; ** P < 0.01; *** P < 0.001; **** P < 0.0001.

Given the fact that γδT17 cells have enriched lipid metabolism and storage, and PRDM16 is a well-known regulator for lipid metabolism, our data imply that PRDM16 could also affect the fitness of γδT17 cells in lipid environments. To investigate this possibility, we compared the expression of genes related to lipid metabolisms between control and *Prdm16* cKO γδT cells. In line with our previous data (**Figures 2H–K**), the loss of Prdm16 caused an increased expression of genes related to lipid metabolisms in γδT17 cells such as Pparg and Cpt1a, with an insignificant increase in Srebp2 and Cd36 expression. (**Supplementary Figures 5A-G**). This suggest that *Prdm16-*deficient γδT17 cells are more likely to adapt in high-lipid environment. When control and *Prdm16* cKO mice were fed with HFD, a marked decrease in body weight and size were observed in *Prdm16* cKO mice compared to that in control mice (**Figures 4A, B**). Specifically, the size and weight of the liver and WAT were greatly decreased in *Prdm16* cKO mice (**Figures 4C, D**). In addition, lipid content, defined by the levels of free fatty acid and triglyceride in the serum of mice, was significantly increased in *Prdm16* cKO mice (**Figures 4E, F**). These results suggest that the loss of *Prdm16* in γδT cells resulted in an increased lipolysis and degeneration of adipose tissue. It has been reported that IL-17 inhibits adipogenesis by suppressing adipocyte differentiation through decreased expression of proadipogenic transcription factors (27), and that IL-17 from adipose γδT17 is crucial for the regulation of adipose tissue (13). Based on these, we compared the phenotypes of adipose γδT17 cells between control and *Prdm16* cKO mice. As expected, the number of γδT17 cells was increased in the liver and epididymal WAT with an insignificant increase in subcutaneous and mesenteric WAT of *Prdm16* cKO mice (**Figure 4G**). In addition, the capacity of IL-17 secretion was greatly increased due to *Prdm16* deficiency (**Figures 4H, I**). These data imply that loss of *Prdm16* increases fitness of γδT17 cells, as well as their function, in a high-lipid environment.

### Loss of *Prdm16* promotes γδT17 cell differentiation and exacerbates skin psoriasis

**Figure 5:**
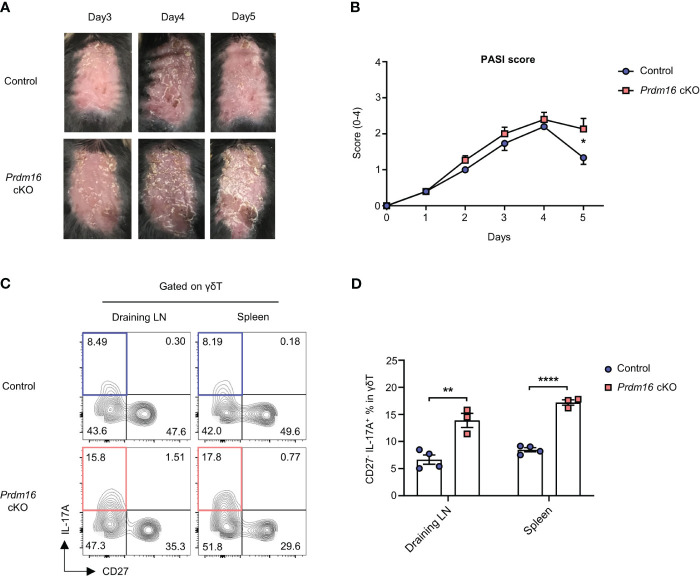
Loss of *Prdm16* promotes γδT17 cell differentiation and exacerbates skin psoriasis. **(A-D)** Imiquimod was applied to the shaved back of control and *Prdm16* cKO mice for 5 consecutive days in order to induce psoriasis. **(A)** The picture of shaved back from control and *Prdm16* cKO mice. **(B)** PASI (Psoriasis Area Severity Index) score of control and *Prdm16* cKO mice (n=5 per group, N=5). (**C**, **D**) Lymphocytes of draining lymph nodes (LN) and spleen from control and *Prdm16* cKO mice were isolated and treated with PMA/Ionomycin. **(C)** Representative flow cytometry plot of IL-17A and CD27 expression in γδT cells. **(D)** The proportion of CD27- IL-17A+ γδT17 cells in γδT cells (n=3-4 per group, N=3). Data are mean ± SEM. Statistical analysis was performed using Student’s *t*-test. * P < 0.05; ** P < 0.01; **** P < 0.0001.

Among the organs, skin is the largest organ with high lipids. Interestingly, lipid content even increases in skin lesion of psoriasis patient and psoriatic mice models (28–30) and it is well known that γδT17 cells are the main driver of skin psoriasis (31). Therefore, there is a high chance that loss of *Prdm16* could affect the pathogenesis of skin psoriasis. When we induced psoriasis by applying imiquimod on the back of mice, the severity score of psoriasis was significantly higher in Prdm16 cKO mice compared to control mice on day 5 (**Figures 5A, B**). Moreover, the proportion of IL-17A secreting CD27^-^ γδT17 cells markedly increased in draining lymph nodes and spleen of *Prdm16* cKO mice (**Figures 5C, D**). These results support that loss of *Prdm16* promotes γδT17 cell differentiation, which in turn exacerbates skin psoriasis.

### *Prdm16* controls lipid-mediated differentiation of Vγ4^+^ γδT17 cells

**Figure 6:**
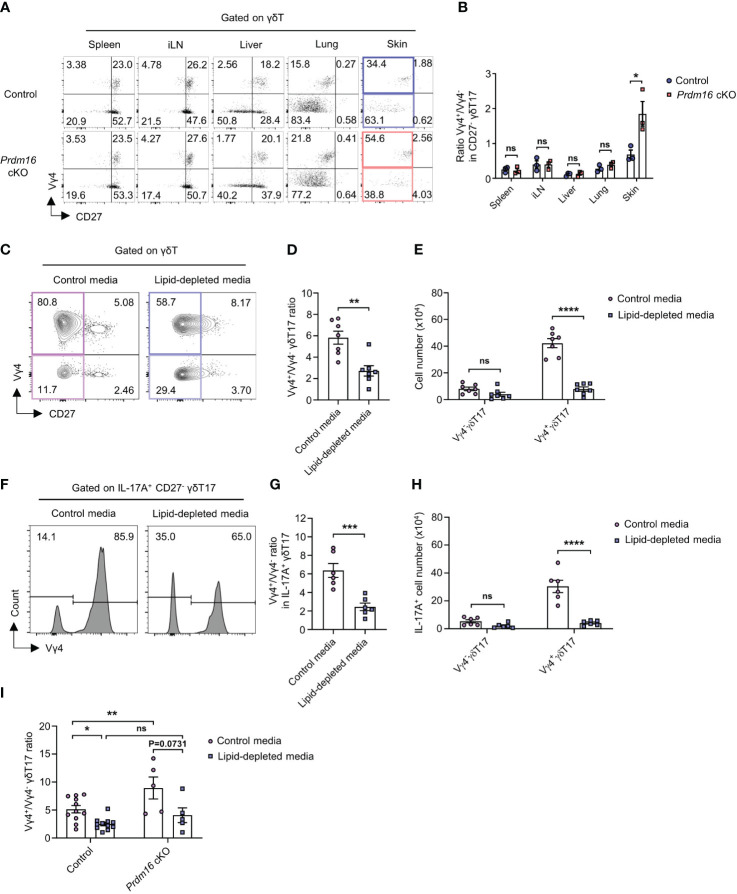
*Prdm16* controls lipid-mediated differentiation of Vγ4^+^ γδT17 cells. **(A, B)** Profiles of γδT cells in various organs from control and *Prdm16* cKO mice were analyzed using flow cytometry. **(A)** Representative flow cytometry plot of Vγ4 and CD27 expression in γδT cells. **(B)** The ratio of Vγ4^+^/Vγ4^-^ in CD27^-^ γδT17 cells (n=3 per group, N=3). **(C-I)** γδT17 cells were generated from spleen lymphocytes under the γδT17 driving condition and γδ TCR stimulation. γδT17 cells were cultured in control media or lipid-depleted media. **(C)** Representative flow cytometry plot of Vγ4 and CD27 expression in γδT cells. **(D)** The ratio of Vγ4^+^/Vγ4^-^ in CD27^-^ γδT17 cells (n=7 per group, N=4). **(E)** Total cell number of Vγ4^-^ γδT17 and Vγ4^+^ γδT17 cells under the control media and lipid-depleted media condition (n=7 per group, N=4). **(F-H)** The cells were treated with PMA/Ionomycin and analyzed using flow cytometry. **(F)** Representative flow cytometry plot of Vγ4 expression in IL-17A^+^ CD27^-^ γδT17 cells. **(G)** The ratio of Vγ4^+^/Vγ4^-^ in IL-17A^+^ CD27^-^ γδT17 cells (n=6 per group, N=4). **(H)** Total cell number of IL-17A^+^ Vγ4^-^ γδT17 and Vγ4^+^ γδT17 cells (n=6 per group, N=4). **(I)** The ratio of Vγ4^+^/Vγ4^-^ in CD27^-^ γδT17 cells from control (n=11) and *Prdm16* cKO (n=5) mice (N=5). Data are mean ± SEM. Statistical analysis was performed using Student’s *t*-test. ns(non-significant); * P < 0.05; ** P < 0.01; *** P < 0.001; **** P < 0.0001.

It is well known that Vγ4^+^ γδT17 cells are mainly responsible for psoriasis among all γδT17 cell subtypes (9). Vγ4^+^ γδT17 cells are known as the major source of IL-17 during the onset of psoriasis (32, 33). Since we observed exacerbated psoriasis in *Prdm16* cKO mice, we hypothesized that *Prdm16* might control the differentiation of Vγ4^+^ γδT17 cells. When we compared Vγ4 chain usage within γδT cells from various immune organs, the proportion of Vγ4^+^ γδT17 cells increased only in the skin of *Prdm16* cKO mice whereas the proportion was comparable in other organs between control and *Prdm16* cKO mice (**Figures 6A, B**). In addition, loss of *Prdm16* enhanced Vγ4^+^ γδT17 cell differentiation *in vitro* as well (**Supplementary Figure 6**). As previously reported, γδT17 cells were prominent among all γδT cells in barrier organs, such as the lung and skin (**Figure 6A**). However, it is notable that distinct phenotypes induced by *Prdm16* cKO occurred only in the skin, which is a lipid-rich organ. These results suggest that PRDM16 regulates the differentiation of Vγ4^+^ γδT17 cells in skin.

Given that lipid content increases in psoriatic skin (28, 29), we speculated that increased lipid might increase Vγ4^+^ γδT17 cell differentiation. To figure out whether lipid itself could affect the differentiation of γδT17 cells, we used lipid-depleted media for *in vitro* γδT17 cell culture. Since control media contain an essential amount of lipids for cell activities, Vγ4^+^ γδT17 cells were the dominant population under type 17 driving condition in control media. However, the proportion of Vγ4^+^ γδT17 cells significantly decreased when they were cultured in lipid-depleted media (**Figures 6C, D**). Although the proportion of Vγ4^-^ γδT17 cells was increased by lipid depletion, cell number count showed that Vγ4^+^ γδT17 cells, not Vγ4^-^ γδT17 cells, were considerably affected by the changes in the lipid content (**Figure 6E**). Also, the proportion and number of IL-17A secreting Vγ4^+^ γδT17 cells were greatly decreased by lipid depletion (**Figures 6F–H**). In line with our data (**Figure 6A**), Vγ4^+^/Vγ4^-^ γδT17 ratio was significantly increased by *Prdm16* deficiency when the cells were cultured in control media. However, the increased ratio by *Prdm16* deficiency was not observed when the cells were cultured in lipid-depleted media (**Figure 6I**). Collectively, these data indicate that the control of Vγ4^+^ γδT17 cell differentiation by *Prdm16* downregulation is largely dependent on lipids.

## Discussion

It is known that γδT17 cells are the primary source of IL-17 secretion in lipid-rich organs like adipose tissue and skin (6, 13, 31). The prospective relationship between γδT17 cells and lipids, such as enriched lipid storage and lipid metabolism in γδT17 cells, has previously been reported (14, 15); however, the molecular mechanism underlying the role of lipid in differentiation and function of γδT17 cells remains elusive. In this study, we showed that loss of PRDM16 promotes γδT17 cell differentiation via upregulating type 17 programs and lipid-dependent cell fitness. PRDM16 is known to negatively regulates lipid storage within a cell by inhibiting adipogenesis or promoting thermogenesis (24, 25). In line with such a negative correlation between PRDM16 and lipid storage in adipocytes, we showed that γδT17 cells, which are known to have high lipid accumulation, display a low *Prdm16* expression. In addition, the expression of *Prdm16* in precursor γδT cells was downregulated by lipids present in ACM, whereas the genes related to lipid metabolic pathways were upregulated. These data indicate that precursor γδT cells can detect the presence of lipid in the environment and rapidly downregulate *Prdm16* to improve lipid-dependent cell fitness required for differentiation into γδT17 cells. Indeed, we demonstrated that the loss of *Prdm16* increased the expression of genes related to lipid metabolism and promoted the expansion of γδT17 cells with a higher capacity for secreting IL-17. These results suggest that γδT17 cells naturally adapt to lipid-rich environment and take advantage of the situation for IL-17 secretion. It is interesting that the effect of *Prdm16* deficiecny is profound only in γδT17 cells even though the expression level is higher in γδT1. Considering this in terms of adipose tissue context, the effect of PRDM16 deletion may be the enhanced function of accepting lipid content for metabolic adaptation. Thus, γδT17 cells, which require lipids for their function, are more likely to be affected by the PRDM16 deletion. However, since γδT1 cells appear not to require lipids for their usual function, the effect of PRDM16 deletion may be less likely to affect their phenotypes. Additionally, we observed that *Prdm16* cKO mice display reduced weight-gain under high fat diet. It has been reported that IL-17 can suppress adipocyte differentiation (27). Since *Prdm16* deficiency greatly increased IL-17 secretion in γδT17 cells and the number of γδT17 cells in adipose tissue, reduced weight-gain in *Prdm16* cKO mice can be explained by these results. However, it is not clear at this point whether IL-17 is the direct cause for the reduced weight-gain. Further investigation using *Il17* and *Tcrd* KO mice would be helpful to resolve this issue.

γδT17 cell is well-known for its anti-microbial immunity and its contribution is most predominant in skin, which serves as a first-line defense barrier (6). Basically, IL-17 secreted by the skin γδT17 cells plays a crucial role in maintaining barrier function against extracellular bacteria by promoting the proliferation and activation of keratinocytes. However, abnormal proliferation and activation of keratinocytes by IL-17 may result in psoriasis. Thus, skin γδT17 cell plays a pivotal role in both bacterial infection and psoriasis (34). Skin is an organ with a layer of lipids; dermal adipocytes proliferate and fat layer of the dermis thickens during bacterial infection (16). Of note, lipids are enriched in psoriatic skin (28–30), and increased adiposity and weight gain are strong risk factors for incident psoriasis (35, 36). We have shown that factors driving type 17 immunity, such as TLR ligands and IL-1β/IL-23 as well as enriched lipids, directly downregulate *Prdm16* expression in γδT cells (**Supplementary Figures 7A, B**). This implies that this phenomenon is highly relevant to the lipid-rich skin where bacterial infection is constantly occurring. Considering that the loss of *Prdm16* increases lipid-dependent cell fitness and γδT17 differentiation, skin γδT cells may evolve to secrete IL-17 for protection against bacterial infection by downregulating *Prdm16* in lipid-rich skin, and excessive secretion of IL-17 may cause the development of psoriasis. Supporting this, we showed that *Prdm16* deficiency was associated with pathogenesis of severe psoriasis in mice. However, it is not clear whether precursor γδT cells exist and differentiate to γδT17 in the skin.

It has been shown that the generation γδT17 cells expressing the Vγ4 TCR chain are largely restricted to fetal embryonic wave (8). It has also been reported that adult thymus-generated c-Maf^+^ RORγt^+^ Vγ4 γδT17 cells fail to reach the periphery (37). However, Vγ4^+^ extrathymic precursor cells (CD27^+^ CD122^-^) in bone marrow/spleen/lymph node have been identified and can differentiate into γδT17 cells upon inflammatory conditions such as EAE and psoriasis (9, 10). Vγ4^+^ γδT17 cells are the dominant IL-17-secreting cell population in psoriatic skin (32). It has been known that dermal Vγ4^+^ γδT17 cells migrate from inflamed skin to draining lymph nodes during psoriasis, proliferate, and then migrate back to the original inflamed tissue (33). However, it remained elusive how the generation of Vγ4^+^ γδT17 cells are controlled during psoriasis. We demonstrated that Vγ4^+^ γδT17 cell differentiation was specifically affected by lipids. The proportion of Vγ4^+^ γδT17 cells was significantly increased by *Prdm16* deficiency only in the skin, a lipid-rich organ. Given that Vγ4^+^ γδT17 cells are the major source of IL-17 during psoriasis, this could also explain the exacerbation of psoriasis in *Prdm16* cKO mice. It has been known that high fat diet exacerbates murine psoriasis by increasing Vγ4^+^ γδT17 cells (12). This result also supports our findings that the generation of Vγ4^+^ γδT17 cells is largely affected by lipids. We observed increased γδT17 cells in adipose tissue of *Prdm16* cKO mice upon high fat diet. For better understanding of Vγ4^+^ γδT17 cell expansion by *Prdm16*, further study on the impact of high fat diet is imperative. Lastly, we have shown that the differentiation of Vγ4^+^ γδT17 cells was greatly diminished by lipid depletion. Thus, our results indicate that lipid itself and lipid-dependent cell fitness are crucial factors for extrathymic differentiation of Vγ4^+^ γδT17 cells, and loss of *Prdm16* further promotes it. However, we cannot exclude completely the possibility that *Prdm16* might regulate the fetal generation wave of Vγ4^+^ γδT cells.

In this study, we have shown that *Prdm16* controls differentiation of γδT cells into γδT17 cells. Factors such as TLR ligands and IL-1β/IL-23 as well as enriched lipids, directly downregulate *Prdm16* expression in γδT cells. Decreased expression of *Prdm16* promotes type 17 immunity gene expression program and lipid-dependent cell fitness, which in turn increases the generation of inducible Vγ4^+^ γδT17 cells that can respond to infection. However, if it goes too extreme, psoriasis might occur (**Supplementary Figure 7C**). Our results suggest that the pathology of γδT cell-derived IL-17-mediated diseases could be regulated by controlling *Prdm16* expression.

## Data availability statement

The datasets presented in this study can be found in online repositories. The names of the repository/repositories and accession number(s) can be found in the article/**Supplementary Material**.

## Ethics statement

The animal study was approved by Institutional Animal Care and Use Committees (IACUC) of Seoul National University. The study was conducted in accordance with the local legislation and institutional requirements.

## Author contributions

JN: Conceptualization, Data curation, Formal analysis, Investigation, Methodology, Visualization, Writing – original draft. YL: Investigation, Methodology, Writing – review & editing. RS: Conceptualization, Funding acquisition, Project administration, Resources, Supervision, Writing – review & editing.
