# Supplementary material for: PRDM16 regulates γδT17 cell differentiation via controlling type 17 program and lipid-dependent cell fitness
Source: Front Immunol. 2024 Jan 4;14:1332386. doi: 10.3389/fimmu.2023.1332386 (PMC10794300; doi:10.3389/fimmu.2023.1332386)
Supplement: Supplementary file 1 [file DataSheet_1.pdf]

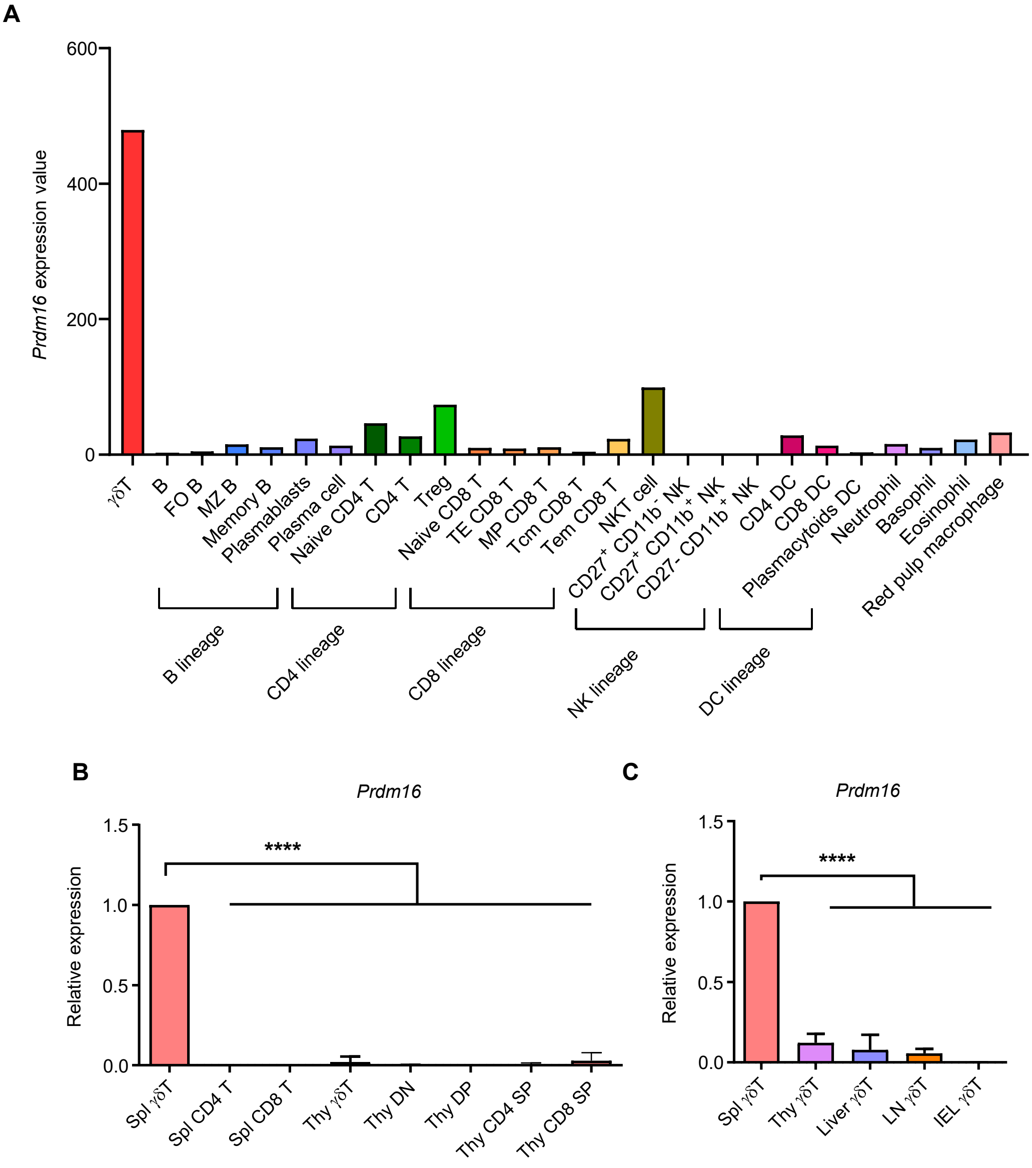

Supplementary Figure 1. ***Prdm16*** **is highly expressed in γδT1 and downregulated in γδT17 cells.** (**A**) Gene expression of *Prdm16* in various immune cells. Data was obtained from ImmGen Project (GSE109125). (**B**) Gene expression of *Prdm16* in T cell populations from spleen and thymus (n=3 per group, N=3). (**C**) Gene expression of *Prdm16* in γδT cells from various organs (n=3~5 per group, N=3). Data are mean±SD. Statistical analysis was performed using one-way ANOVA followed by Tukey's multiple comparisons test. **** $P < 0.0001$.

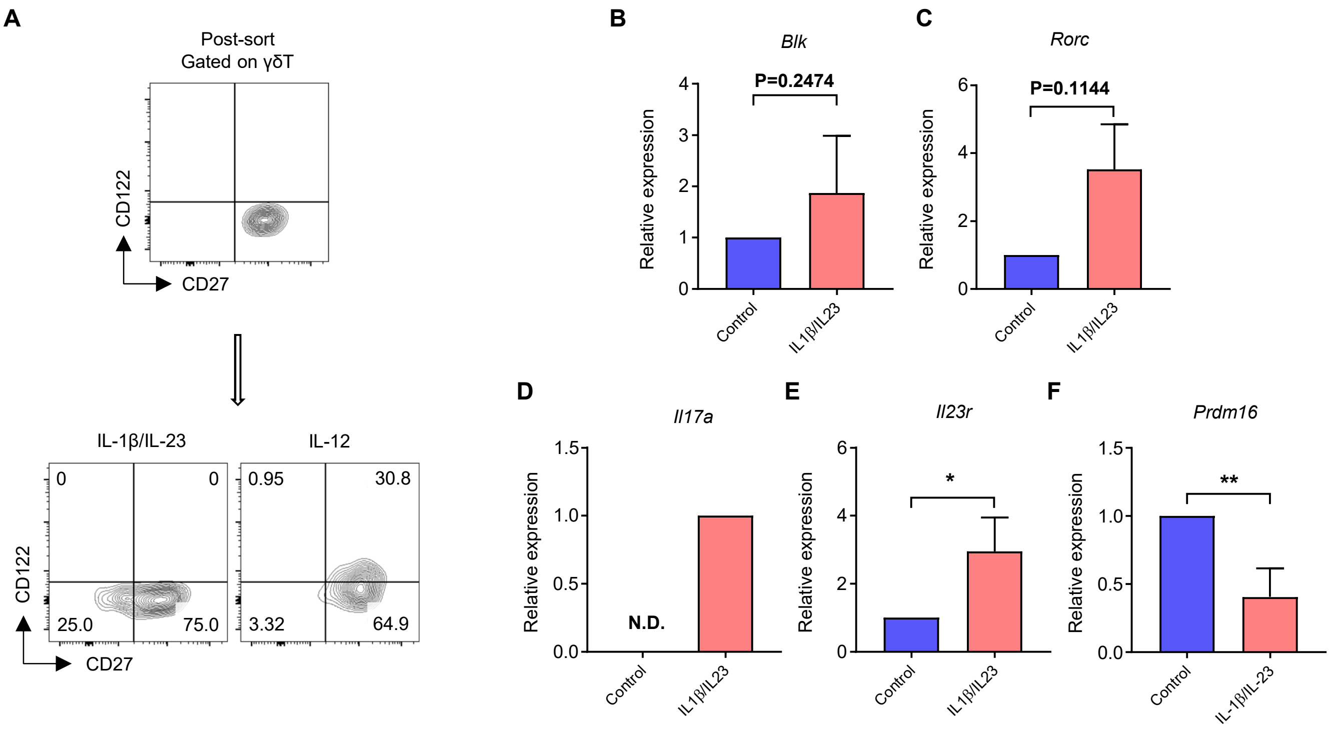

Supplementary Figure 2. ***Prdm16*** **is rapidly downregulated in response to γδT17 cell-driving cytokines.** (**A**) Precursor γδT ($CD27^+$ $CD122^-$) cells were isolated and cultured under γδT1 cell-driving and γδT17 cell-driving condition. Representative flow cytometry plot of CD122 and CD27 expression in γδT cells. (**B**-**F**) Precursor γδT ($CD27^+$ $CD122^-$) cells were isolated and treated with γδT17 cell-driving cytokines (IL-1β and IL-23) for 4hrs. Gene expression profile was analyzed by qPCR. mRNA expression of (**B**) *Blk* (n=3 per group, N=3), (**C**) *Rorc* (n=3 per group, N=3), (**D**) *Il17a* (n=3 per group, N=3), (**E**) *Il23r* (n=3 per group, N=3), (**F**) *Prdm16* (n=3 per group, N=3). Data are mean±SD. Statistical analysis was performed using Student's *t*-test. ns(non-significant); *P < 0.05; ** P < 0.01.

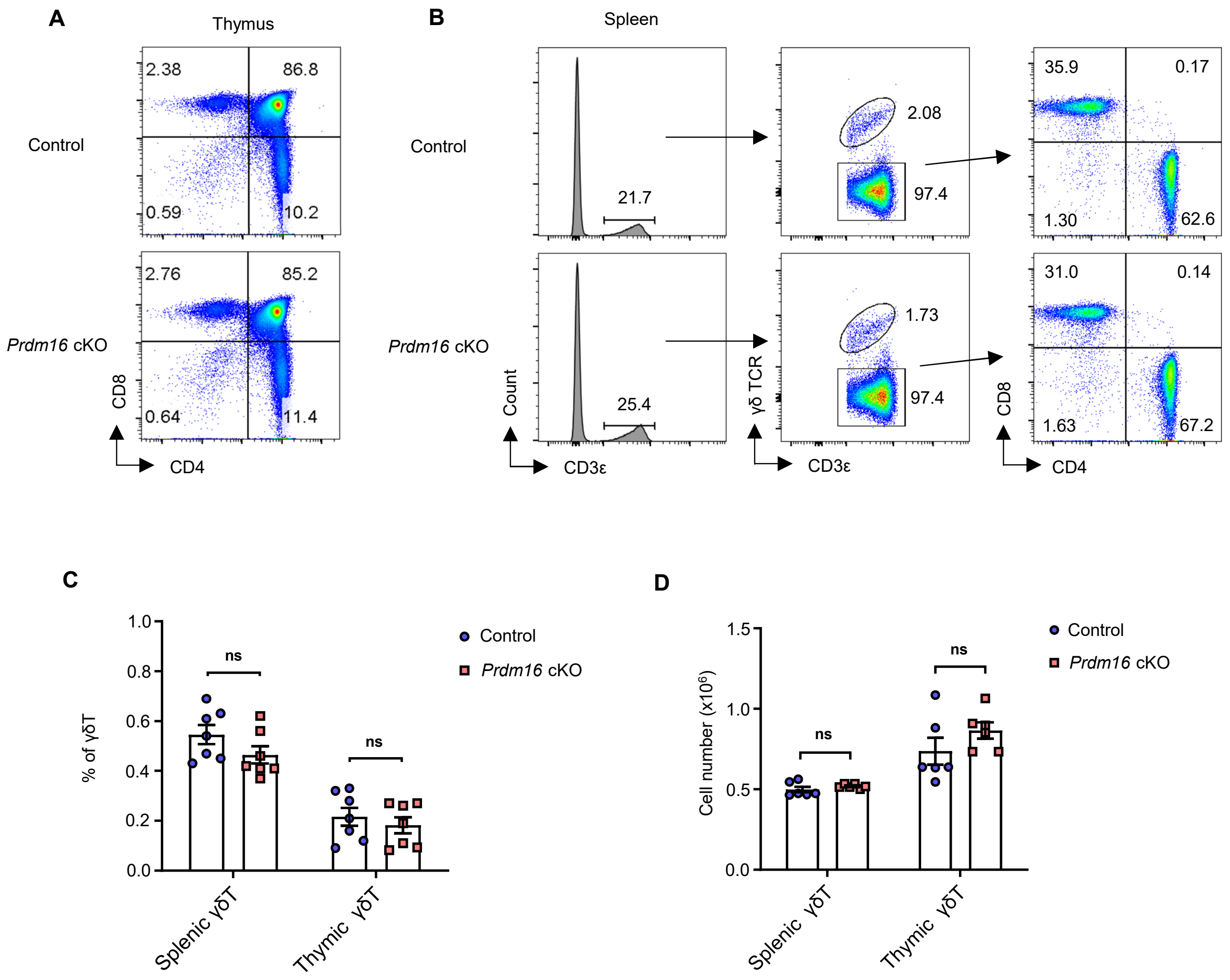

Supplementary Figure 3. Normal development of T cell lineages in the thymus and spleen of *Prdm16* cKO mice. (**A-D**) T cell lineages were analyzed in the thymus and spleen of control and *Prdm16* cKO mice using flow cytometry. (**A**) Representative flow cytometry plot of CD4 and CD8 expression in thymic lymphocytes. (**B**) Representative flow cytometry plot of CD3ε, CD4 and CD8 in splenic lymphocytes. (**C**) The proportion of γδT cells in spleen and thymus (n=7 per group, N=4). (**D**) Total cell number of γδT cells in spleen and thymus (n=6 per group, N=4). Data are mean±SEM. Statistical analysis was performed using Student's *t*-test. ns(non-significant).

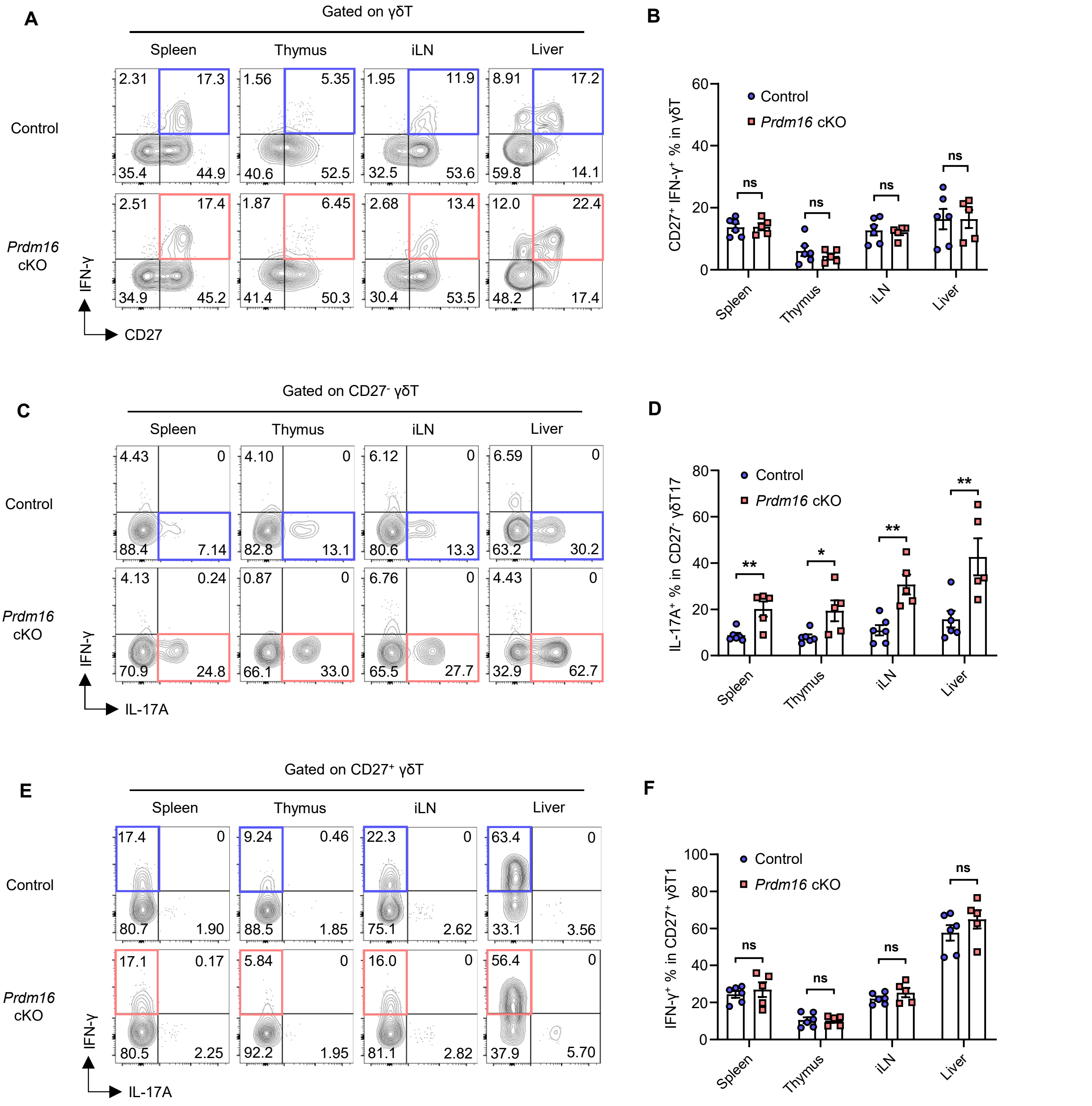

Supplementary Figure 4. Loss of *Prdm16* enhances IL-17A secretion in γδT17 cells. (**A**-**F**) Lymphocytes of various organs (spleen, thymus, iLN, liver) from control and *Prdm16* cKO mice were isolated and treated with PMA/Ionomycin. Cytokine secretion was analyzed using flow cytometry. (**A**) Representative flow cytometry plot of IFN-γ and CD27 expression in γδT cells. (**B**) The proportion of $CD27^{+}$ IFN-γ^{+} cells in γδT cells (n=5-6 per group, N=4). (**C**) Representative flow cytometry plot of IFN-γ and IL-17A expression in $CD27^{-}$ γδT (γδT17) cells. (**D**) The proportion of IL-$17A^{+}$ cells in $CD27^{-}$ γδT (γδT17) cells (n=5-6 per group, N=4). (**E**) Representative flow cytometry plot of IFN-γ and IL-17A expression in $CD27^{+}$ γδT (γδT1) cells. (**F**) The proportion of IFN-γ^{+} cells in $CD27^{+}$ γδT (γδT1) cells (n=5-6 per group, N=4). Data are mean±SEM. Statistical analysis was performed using Student's *t*-test. ns(non-significant); * $P < 0.05$; ** $P < 0.01$.

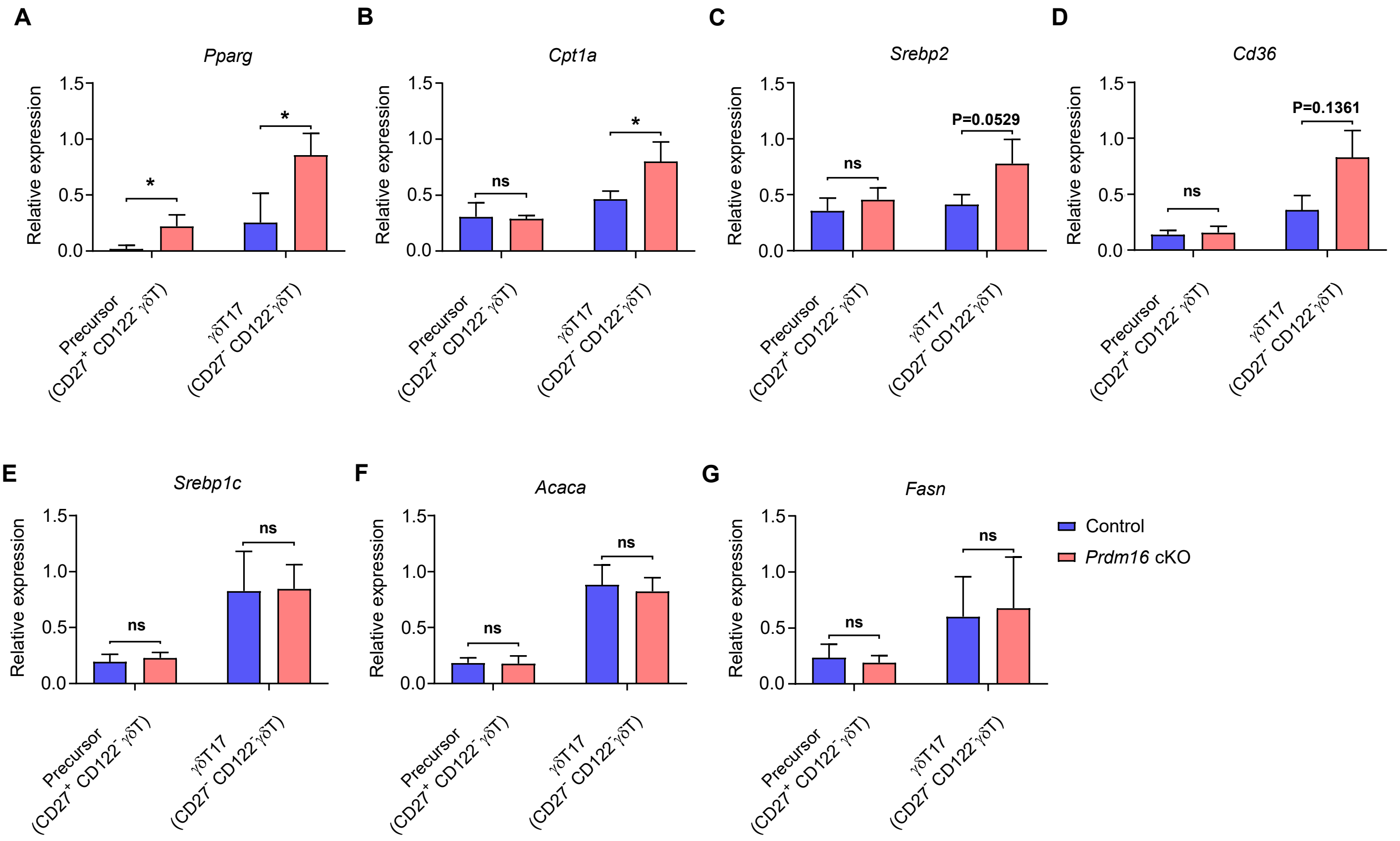

Supplementary Figure 5. Loss of *Prdm16* increases the expression of genes related to lipid metabolism in γδT17 cells. (**A-G**) Precursor γδT ($CD27^{+}$ $CD122^{-}$) cells and γδT17 ($CD27^{-}$ $CD122^{-}$) cells were isolated from spleen of control and *Prdm16* cKO mice. The expression of genes related lipid metabolism was analyzed by qPCR. mRNA expression of (**A**) *Pparg* (n=3 per group, N=3), (**B**) *Cpt1a* (n=3 per group, N=3), (**C**) *Srebp2* (n=3~4 per group, N=3), (**D**) *Cd36* (n=3 per group, N=3), (**E**) *Srebp1* (n=3 per group, N=3), (**F**) *Acaca* (n=3~4 per group, N=3), (**G**) *Fasn* (n=3~4 per group, N=3). Data are mean±SD. Statistical analysis was performed using Student's *t*-test. ns(non-significant); * $P < 0.05$.

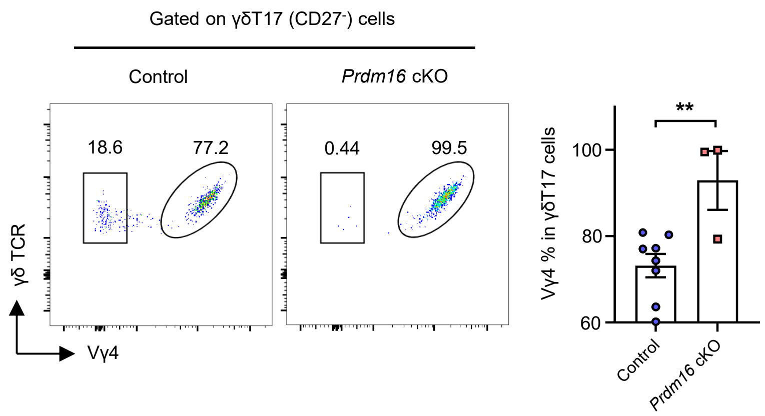

Supplementary Figure 6. Loss of *Prdm16* promotes Vγ4⁺ γδT17 cell differentiation *in vitro*. γδT17 cells were generated from spleen lymphocytes of control and *Prdm16* cKO mice under the γδT17 cell driving condition and γδ TCR stimulation. Representative flow cytometry plot and the proportion of $V\gamma4^{+}$ $CD27^{-}$ γδT17 cells (n=3-8 per group, N=3). Data are mean±SEM. Statistical analysis was performed using Student's *t*-test. ** $P < 0.01$.

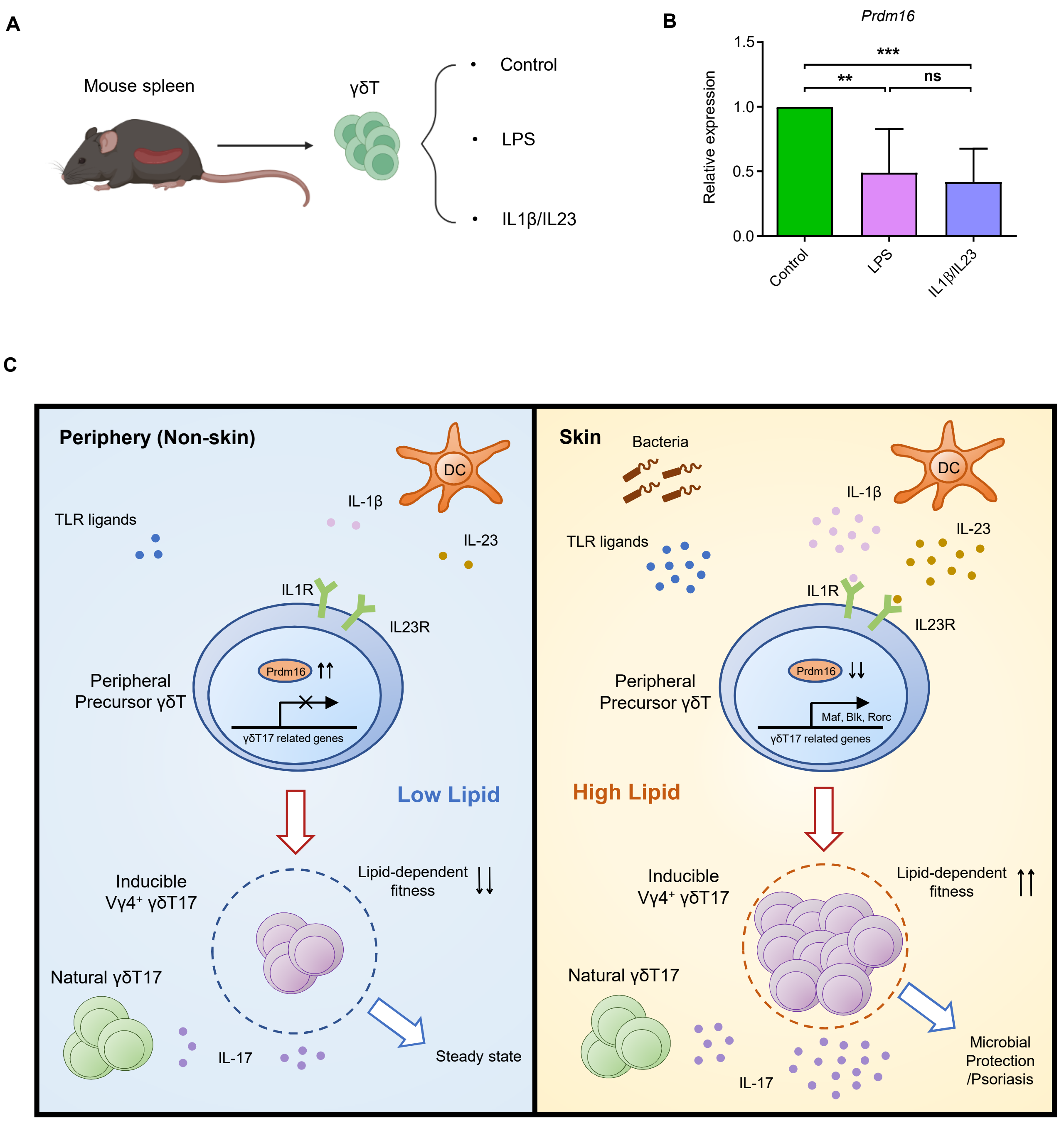

Supplementary Figure 7. Enriched lipids, along with type 17 immunity driving factors, downregulate *Prdm16* expression, promoting the generation of inducible $V\gamma4^{+}$ γδT17 cells in skin. (**A** and **B**) γδT cells were isolated from C57BL/6 mouse spleen and were treated with LPS and IL-1β/IL-23 for 4hrs. (**A**) Schematic design of the experiment. (**B**) mRNA expression of *Prdm16* in control, LPS, and IL-1β/IL-23 treated γδT cells (n=7 per group, N=5). Data are mean±SD. Statistical analysis was performed using Student's *t*-test. ns(non-significant); ** $P < 0.01$; *** $P < 0.001$. (**C**) Schematic model highlighting the role of PRDM16 in γδT cells

#Primers for qRT-PCR	
Actb Forward	CGTGAAAAGATGACCCAGATCA
Actb Reverse	TGGTACGACCAGAGGCATACAG
Blk Forward	TTATGTGCCCAGCAACTTTGTG
Blk Reverse	AAGGCACCTTTATTGCTCTCACTCT
Rorc Forward	TTTGGAACTGGCTTTCCATC
Rorc Reverse	AAGATCTGCAGCTTTTCCACA
Maf Forward	AGCAGGTAGACCACCTCAAGCA
Maf Reverse	GAGTCCCTTGGGTACATGAAAAATT
Tbx21 Forward	AGCAAGGACGGCGAATGTT
Tbx21 Reverse	GGGTGGACATATAAGCGGTTC
Eomes Forward	CGTTCACCCAGAATCTCCTAACA
Eomes Reverse	TGCAGCCTCGGTTGGTATTT
Ifng Forward	GATGCATTCATGAGTATTGCCAAGT
Ifng Reverse	GTGGACCACTCGGATGAGCTC
Prdm16 Forward	CAGCACGGTGAAGCCATTC
Prdm16 Reverse	GCGTGCATCCGCTTGTG
Pparg Forward	GTGCCAGTTTCGATCCGTAG
Pparg Reverse	GGCCAGCATCGTGTAGATGA
Cd36 Forward	ATTGGTCAAGCCAGCT
Cd36 Reverse	TGTAGGCTCATCCACTAC
Cpt1a Forward	CCAGGCTACAGTGGGACATT
Cpt1a Reverse	GAACTTGCCCATGTCCTTGT
Il17a Forward	GGCCCTCAGACTACCTCAAC
Il17a Reverse	TCTCGACCCTGAAAGTGAAGG
Il23r Forward	TTCAGATGGGCATGAATGTTTCT
Il23r Reverse	CCAAATCCGAGCTGTTGTTCTAT
Srebp2 Forward	CCCTTGACTTCCTTGCTGCA
Srebp2 Reverse	GCGTGAGTGTGGGCGAATC
Srebp1c Forward	GGAGCCATGGATTGCACATT
Srebp1c Reverse	GGCCCGGGAAGTCACTGT
Acaca Forward	CCTCCGTCAGCTCAGATACA
Acaca Reverse	TTTACTAGGTGCAAGCCAGACA
Fasn Forward	GGTCGTTTCTCCATTAAATTCTCAT
Fasn Reverse	CTAGAAACTTTCCCAGAAATCTTCC

Supplementary Table 1. Primer list for qRT-PCR.
